# Lung Cancer Risk and Low (≤50 μg/L) Drinking Water Arsenic Levels for US Counties (2009–2013)—A Negative Association

**DOI:** 10.3390/ijerph15061200

**Published:** 2018-06-07

**Authors:** Steven H. Lamm, Isabella J. Boroje, Hamid Ferdosi, Jaeil Ahn

**Affiliations:** 1 Center for Epidemiology and Environmental Health (CEOH, LLC), Washington, DC 20016, USA; Borojej@gmail.com (I.J.B.); Hamid@CEOH.com (H.F.); 2 Department of Health Policy and Management, Bloomberg School of Public Health, Johns Hopkins University, Baltimore, MD 21205, USA; 3 Department of Pediatrics, Georgetown University School of Medicine, Washington, DC 20007, USA; 4 Milken Institute School of Public Health, George Washington University, Washington, DC 20052, USA; 5 Department of Biostatistics, Bioinformatics, and Biomathematics, Georgetown University School of Medicine, Washington, DC 20007, USA; ja1030@georgetown.edu

**Keywords:** arsenic, lung cancer, drinking water, dose-response, risk

## Abstract

While epidemiologic studies clearly demonstrate drinking water with high levels of arsenic as a significant risk factor for lung cancer, the evidence at low levels (≤50 μg/L) is uncertain. Therefore, we have conducted an ecological analysis of recent lung cancer incidence for US counties with a groundwater supply of <50 μg/L, the historical limit for both the EPA and WHO. Data sources used included USGS for arsenic exposure, NCI for lung cancer outcome, and CDC and US Census Bureau forcovariates. Poisson log-linear models were conducted for male, female, and total populations using for exposure median county arsenic level, maximum arsenic level ≤50 μg/L, and ≥80% population groundwater dependency. Statistically significant negative associations were found in each of the six models in which the exposure was limited to those who had major exposure (≥80% dependency) to low-levels of arsenic (≤50 μg/L). This is the first large ecological study of lung cancer risk from drinking water arsenic levels that specifically examined the dose-response slope for populations whose exposure was below the historical limit of ≤50 μg/L. The models for each of the three populations (total; male; female) demonstrated an association that is both negative and statistically significant.

## 1. Introduction

Arsenic levels naturally found in groundwater range from μg/L to mg/L throughout the world. Major epidemiological studies from areas with very high arsenic exposures measured in hundreds of μg/L to a few mg/L, particularly in Taiwan, Southeast Asia, and South America, have been conducted and have demonstrated increased cancer risk at exposure levels of 300–2000 μg/L. The first showing for lung cancer excess was from the Blackfoot-Disease endemic area of Southwest Taiwan [1,2] with increased risk for the villages with median exposures between 300 μg/L and 600 μg/L and still higher for those with median exposures above 600 μg/L. Similarly, studies from Chile showed that their increased risk was among those with arsenic exposures of 800 μg/L or greater [3]. Additionally, some studies purportedly showed an increase at exposures in the 100–300 μg/L range [4,5]. 

The dose-response relationship for arsenic concentrations below 100 μg/L is uncertain. Some studies reported a positive relationship [6,7], some no relationship [8], and some a negative relationship [9]. Both D’Ippolito (2015) [6] and Ferdosi (2016) [8] were mortality studies with mean and median, respectively, exposures up to 60 μg/L. Both Mendez (2017) [7] and Dauphine (2013) [9] were incidence studies with mean exposures up to 50 μg/L and 110 μg/L, respectively, with occasional cases having much higher levels.

Historically, allowable levels of arsenic in drinking water had been set at ≤50 μg/L by the US Public Health Service in 1942, the World Health Organization (WHO) in 1963, and the US Environmental Protection Agency (US EPA) in 1975 [10]. These limits had been based on the sensitivity of analytic methods and on evidence of non-carcinogenic toxicities at higher levels. However, following the risk assessments of skin cancer studies in Southwest Taiwan [11,12] and later of studies of internal cancers (i.e., bladder and lung) in Southwest Taiwan [2,13,14], stricter limits have been set at 10 μg/L by the WHO in 1993 [10] and, following similar findings in Chile [15], by the US EPA in 2001 with an effective date of 2006 (US EPA 2001) [16]. The risks at these levels have not been previously assessed.

Recognizing that the ≤50 μg/L drinking water arsenic standard, called the Maximum Contaminant Level (MCL), was in effect for over 70 years, we take this opportunity to examine the dose-response relationship for lung cancer incidence for communities that were in compliance of this standard. This inquiry begins with 757 counties for which there are publicly available county-specific arsenic levels in drinking water wells and publicly available county-specific lung cancer incidence data. It then restricts its analysis to the 417 counties whose populations were ≥ 80% dependent on groundwater wells for their drinking water supply and whose drinking water well arsenic levels were known not to exceed 50 μg/L. Thus, the opportunity exists here to examine the arsenic and lung cancer data for risks relevant to compliance to the old 50 μg/L standard and lay the predicate for assessing the new 10 μg/L standard.

## 2. Methods and Materials

This ecological study examines for US counties the direction of the dose-response for lung cancer with respect to the median drinking water arsenic level (μg/L). This study uses two US governmental datasets in the public domain to assess the dose-response relationship between lung cancer incidence (data from the National Cancer Institute, NCI) and arsenic levels of groundwater wells used as drinking water (data from the United States Geological Survey, USGS), using data aggregated at the county level. Additional confounder data came from publicly available US governmental data sets or published data sets. Counties and county-equivalents were identified by their five-digit Federal Information Processing Standard (FIPS) number that had been issued by the National Institute of Standards and Technology (NIST).

The two study criteria for inclusion of US counties in the initial analysis were the public availability of (1) county-specific lung cancer incidence rates for the time-period 2009–2013 and (2) arsenic concentration of groundwater from wells used for drinking water, from which county-specific median arsenic concentrations could be calculated. Lung cancer rates, smoking rates, and demographic variables (where feasible) were obtained for the total population and separately for males and females. Smoking prevalence was the primary confounding variable of interest. Additional county-specific demographic variables, such as residency, education, household income, and poverty, were also obtained. 

### 2.1. Lung Cancer Case Counts

Age-adjusted county-specific lung cancer incidence rates for the period 2009–2013 were obtained from the state cancer profiles website of the NCI [17]. The age-adjusted incidence rates had been adjusted to the 2000 US standard population age-gender distribution and were reported for the total population, for males, and for females based on incidence cases during the five-year interval. Rates were suppressed if the total five-year case count for a particular demographic group was fewer than 16 cases in the five-year period (111 counties) or if public release was not permitted by state administrative or legislative decision (Three states—Kansas, Minnesota, and Nevada).

County-specific lung cancer case counts were calculated as the product of the annual age-adjusted lung cancer incidence rate, the 2010 county population, and the 5-year observation period (2009–2013). 

### 2.2. Groundwater Median Arsenic Concentrations

The National Water-Quality Assessment program of the United States Geological Survey (USGS) released to the public in November, 2001 the National Water Information System(NWIS) dataset that reported the most recent inorganic arsenic measurement (μg/L) of sampled groundwater wells in the US [18]. Arsenic analyses had been performed using either hydride generation or inductively coupled plasma mass spectrometry (ICP/MS). Wells were specified by use—drinking water, non-drinking water (i.e., agricultural or industrial), or unknown—and by which county’s population they supplied. The dataset contained the most recent arsenic measurement (1973–2001) for each of 20,043 individual wells, of which 7287 were identified as drinking water wells. The median groundwater drinking concentration was calculated for each county based on the arsenic data for the wells supplying that county, as previously determined by USGS. Measurements that were below the limit of detection (LOD = 1 μg/L) were entered as 0.5 μg/L (i.e., LOD/2). Three states (Minnesota, Texas, and Wisconsin) and one US EPA Region (Region 1, comprised of the six New England states—Connecticut, Maine, Massachusetts, New Hampshire, Rhode Island, and Vermont) did not consent to the public release of their data.

The median was chosen as the summary metric, rather than the mean, because most counties had very few wells providing their water supply (median = 2). The median provides stability against markedly high measurements that are not provided by the mean. The distinction has to be made between water supplied to a county and water delivered to the county’s residents. Water, whose arsenic levels are much higher than the arsenic standard of <50 μg/L, are unlikely to be delivered to the residents but instead would be substituted with water meeting the arsenic standard. For that reason, and in order to examine the dose-response relationship between lung cancer incidence and the arsenic levels in drinking water compatible with the arsenic standard, analyses for counties that only had drinking water supplies with arsenic levels <50 μg/L arsenic provide the core of this paper.

### 2.3. Dependency of County Population on Groundwater as Source of Drinking Water

The USGS regularly (every five years) releases reports on the estimated water usage by county, which includes data on both populations served and the amount of water withdrawn [19]. The drinking water supplies are categorized as either self-supplied domestic water (i.e., local private wells) or public supply sources, which may be further identified as either from groundwater or from surface water sources. In the years 1985, 1990, and 1995, the report included county-specific data on total population, population using public water from groundwater, population using public water from surface waters, and population using self-supplied waters. 98% of the water from self-supplied sources came from groundwater sources. Additional data included the volume of fresh water and of saline water used by each group. Dependency estimates were limited to the years 1985, 1990, and 1995 as the subsequent reports were incomplete. Unlike the earlier reports, the report for the year 2000 did not separate populations into those using public waters from groundwater sources and those from surface water sources, and the reports for 2005 and 2010 contained such information on only 55% of the counties. 

The degree of dependency on groundwater as the drinking water source of the county’s population was calculated as the sum of the population using public supply water from groundwater sources and the population using self-supplied (usually private wells) domestic water divided by the total population of the county for that year. For the purpose of these analyses, the county-specific drinking water dependency on groundwater was calculated as the average of the percentages for 1985, 1990, and 1995.

### 2.4. Smoking Prevalence Rates

County-specific, gender-specific smoking prevalence rates were obtained from the Small Area Estimates (SAE) for county-related measures developed by the National Cancer Institute (NCI) and derived from two governmental surveys for the period 2008–2010 [20]. These two surveys are the Behavioral Risk Factor Surveillance System (BRFSS) of the Centers for Disease Control and Prevention (CDC, DHHS, USA) and the National Health Interview Survey (NHIS) of the National Center for Health Statistics (NCHS, DHHS, USA). The data were reported as the prevalence of current-smokers and the prevalence of ever-smokers. Ever-smokers were defined as persons 18 years of age or older who reported smoking at least 100 cigarettes in his/her lifetime by the time of interview, and current smokers were defined as those ever-smokers who reported smoking cigarettes some days or every day at the time of interview. The prevalence of ex-smokers was calculated as the prevalence of ever-smokers minus the prevalence of current smokers. The prevalences of current smokers and of ex-smokers were used in these analyses. 

### 2.5. Radon Levels

Radon, after smoking, is the major ubiquitous environmental cause of lung cancer [21]. The US Surgeon-General declared that indoor radon is the second-leading cause of lung cancer in the US [22]. County-specific data from USEPA allows the stratification of counties into those with predicted average indoor radon screening levels greater that the action level of 4 pCi/L and those with lower predicted levels [23].

### 2.6. Demographic Variables

Demographic variables obtained from the 2010 American Fact Finder site of the US Census Bureau included race (White, Black, Asian, Other), ethnicity (Hispanic), educational attainment (completion of high school or equivalency (5-year average)), poverty (proportion below poverty level), residency (proportion living in same county the previous year), and median household income (MHHI ($ K)) [24]. The race and ethnicity data were proportions from the 2010 US census, while the data for the other variables were estimated 5-year averages (2009–2013). County-specific proportions of the 2010 county population that were not urban were obtained from the US Census geo urban area reference website. Obesity prevalence rates by sex were obtained from the CDC’s Diabetes Data and Trends site [25].

### 2.7. Statistical Methods

Linear regression and Poisson log-linear regression models were used to examine the relationship between the median arsenic levels in wells used as a drinking water source for each county and the county’s lung cancer incidence rates. The Poisson log-linear model is formulated as follows,

$$\log\left(\lambda_{c}\right)=\log\left(N_{c}\right)+\beta_{0}+\beta_{1}\times Arsenic\;exposure_{c}+\;\gamma^{T}\times F_{c},$$

where lung cancer incidence (estimate of case count, $\lambda_{c}$) was the dependent variable, and median groundwater arsenic concentration was the primary independent variable. The model offset, log ($N_{c})$, is defined as the natural logarithm of county populations ($N_{c})$. Confounders such as smoking prevalence rates and demographic variables were included as covariates ($F_{c}$) in the adjusted model. The results were statistically significant if a two-sided *p* < 0.05 (Wald *z*-score > 1.96). Both unadjusted and adjusted models were separately run for males, females, and total populations. Stepwise regression was conducted with the elimination of covariates whose p-values were not less than 0.100. The goodness of fit statistics were assessed to check the adequacy of the fitted model, and both Pearson’s and standardized residuals and the quasi-Poisson model were examined for the validity of the Poisson assumption, such as over- and under-dispersion. The outlier and influential observations were investigated using Cook’s distance and delta-betas. Restricted analyses were conducted using the data for the counties for whom the population was more than 80% dependent on groundwater wells for their drinking water supply and whose maximum arsenic levels were ≤50 μg/L. Sensitivity analyses were also performed to evaluate whether estimated associations were sensitive to the filtering of data and choice of model covariables. We used the STATA 15 and R (ver 3.3) statistical packages.

## 3. Results

**Figure 1:**
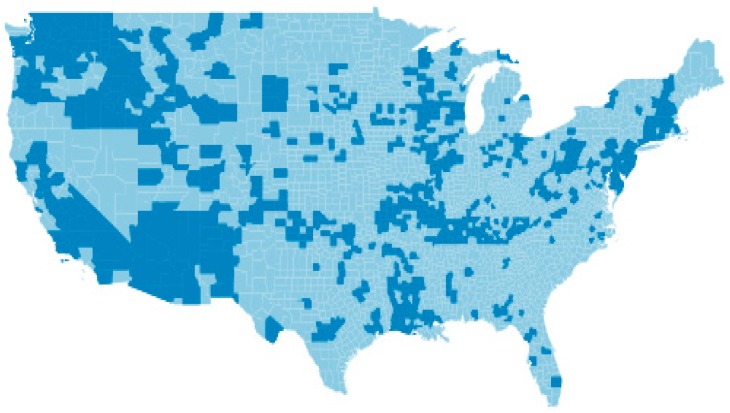
US (48 states) showing counties (in dark) that are included in the analytic dataset.

The master dataset was formed by combining the information of 3147 US counties obtained from the state cancer profiles developed by the NCI and from the NWIS developed by the USGS. Lung cancer incidence rates were available for 2716 counties and groundwater drinking water arsenic levels were available for 757 of these counties. The data for these 757 counties served as the analytic database. Total lung cancer rates were available for all the 757 counties with drinking water arsenic data. Male lung cancer rates were available for 704 counties, and female lung cancer rates were available for 678 counties. The population of these counties comprised 38% of the 2010 US total population (117.2 M/308.7 M = 38%) and nearly 600 million person-years of observation. These counties were in 43 states and distributed within the US as seen in Figure 1. The dark areas are the counties in the analytic set.

### 3.1. Data Characteristics

The data characteristics of the analytic variables for the 757 counties in the analysis are seen in Table 1 for the total county populations. Data characteristics for the male and female populations differed little from those for the total population.

#### 3.1.1. Outcome

Age-adjusted lung cancer incidence rates ranged from 13.5 to 124.8 cases per 100,000 residents per year and varied by only a 10-fold factor. They are normally distributed with kurtosis and skewness each less than 1.0 in all models. Both the mean and median rates were 66.3 cases per 100,000 residents per year. The number of lung cancer cases per county estimated for the five-year (2009–2013) period ranged widely from 7.7 cases to 11,459 cases with a mean count of 463 cases and a median count of 136 cases. This reflects the wide spectrum in county population sizes from about 2.4 thousand to 4.1 million.

#### 3.1.2. Exposure

The proportion of the population having exposure to the drinking water wells ranged from <1% to 100%. Most of the population of these counties were greatly dependent on the groundwater wells as their source for drinking water where the distribution was right skewed with a mean of 74% and a median of 87%. A dependency of 75% or greater was found in 433 counties (57%), and a dependency of less than 25% was found in 66 counties (9%). While some counties had a large number of drinking water wells (range, 1–274), most counties had only a few drinking water wells (median 2). The sampling dates ranged between 1 January 1976 and 30 March 2001 with a median date in 1988 and an interquartile range of 1982 to 1993. Thus, with a median sample date of 1988 and a median diagnosis date of 2011, the date of the exposure measurement data preceded the date of the diagnostic data by more than 23 years, which allows for a lung cancer latency of greater than 20 years.

Arsenic levels in the drinking water ranged between non-detected (limit of detection [LOD] of 1 μg/L) and levels detected up to 950 μg/L. for counties with lung cancer rates, and county median drinking water arsenic levels ranged between non-detected and levels up to 102 μg/L (mean, 2.1; median 0.8). Specimens with no detected arsenic were entered into the analysis as 0.7071 μg/L, i.e., 1/(sqrt 2) × LOD. Most counties (400/757 = 53%) had only 1 or 2 wells as drinking water sources.

#### 3.1.3. Co-Variates

The secondary covariate of interest in any analysis of lung cancer rates is cigarette smoking. The prevalence of current smokers ranged between 6.2 and 40.7% with a mean 24.3% and a median 24.7%. The prevalence of former smokers ranged between 10.6 and 37.4% with a mean 23.1% and a median 23.4%. Ranges for the covariates analyzed are seen in Table 1. For most covariates, the mean and the median were quite similar.

#### 3.1.4. Populations

The proportion of the population that was male ranged between 0.445 and 0.641 with means and medians slightly below 0.500 (mean, 0.498; median 0.495). While the proportion of Hispanics ranged markedly from 0.00 to 0.82, the mean and median were quite low (mean, 0.09; median, 0.04). Similar wide ranges were seen for the other racial groups (White, Black, Asian, and Other) with a high mean and median for Whites (mean, 0.87; median, 0.92) and low means and medians for Blacks, Asians, and Other (mean, 0.07 and 0.02; median, 0.02 and 0.01; and mean, 0.01; median, 0.97, respectively). The major group in the Other was American Indians with the high levels on Indian reservations. The county populations ranged from about 2.4 thousand to about 4.1 million. The large difference between the mean and the median (mean, 154,590; median, 38,966) indicated that the county populations were skewed to the low side. 

### 3.2. Linear Regression Model

**Figure 2:**
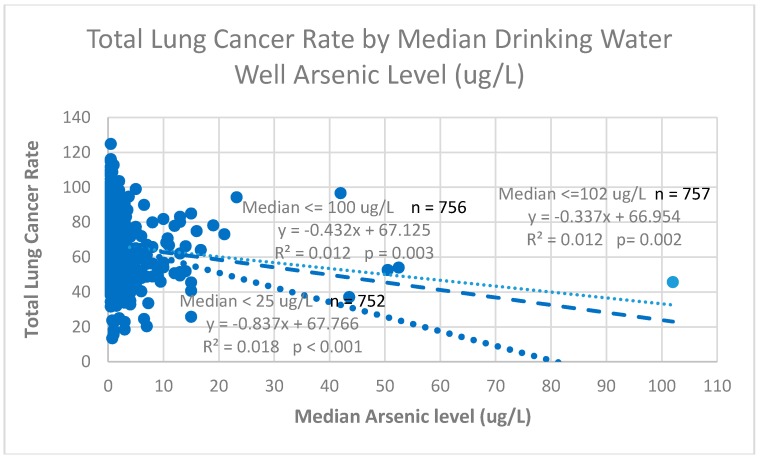
Linear regression of lung cancer rate by median drinking water well arsenic level over three exposure intervals.

Figure 2 shows the distribution of county-specific total lung cancer rates with respect to the county-specific median groundwater well arsenic level used for drinking water.

Overall, the slope for the total lung cancer risk for the total data set with respect to the median arsenic level was negative (−0.337) and statistically significantly (*p* = 0.002) over the approximate range of 0.5 to >100 μg/L. The slope for the male lung cancer risk was also negative (−0.539) and statistically significant (*p* < 0.001), while the slope for the female lung cancer risk was negative (−0.132) but not statistically significant (*p* = 0.146). The R^2^ values indicated that the arsenic exposure explained less than 2% of the variability in the county-specific lung cancer risks. These analyses did not take into consideration either smoking prevalence or the prevalences of other confounders.

One influential observation (median arsenic level of 102) was identified by high Cook’s D values. We re-fitted the model by omitting the observation (Inyo County, CA). We observed that the estimated coefficient changed from −0.337 to −0.432 with the p-value slightly increased from 0.002 to 0.003 for the total lung cancer risk. For female and male lung cancer risks, the estimated coefficients changed to −0.684 and −0.140 with p-values of 0.001 and 0.244, respectively. When we further similarly excluded two potential influential observations (Lake County, MO, and Malheur County, OR) with median arsenic levels of 50.5 and 52.5 and refitted the model, we again had substantially changed coefficients of −0.525 (*p *= 0.004), −0.729 (*p *= 0.005), −0.207 (*p *= 0.151) for the total, male, and female lung cancer risk, while the directions and the significance remained the same. Figure 2 reveals the paucity of counties (*n* = 5; <1%) with median arsenic levels greater than 25 μg/L. The coefficient for the total cancer risk of the 752 counties with median arsenic level <25 μg/L was −0.837 (*p*-value < 0.001) (Figure 2) 

### 3.3. Poisson Log-Linear Regression Model

Table 2 presents the analytic results for the Poisson log-linear regression model for the 757 counties with lung cancer incidence as the dependent variable and median county drinking water arsenic level [As(Median)] as the primary independent variable. The results of both the unadjusted and the covariate-adjusted models are shown for total lung cancer and, separately, for lung cancer in male residents and for lung cancer in female residents. Table 2 gives the log relative risk coefficient (slope) for the median drinking water arsenic levels and covariates with the asterisks indicating the level of statistical significance.

The unadjusted analyses of the county-specific lung cancer incidence (case count estimates) yielded highly statistically significant negative coefficients (*p* < 0.001) for the slope of the median level of arsenic in the drinking water (μg/L) for counties that receive drinking water from groundwater wells in all three study populations (Total, Male, and Female).

In the adjusted analyses, negative coefficients were found in all three population groups and were statistically significantly negative for the total populations (*p* = 0.045) and the male populations (*p* < 0.001) but not for the female populations (*p* = 0.740). The adjusted analyses take into consideration the degree of dependence on groundwater wells as the drinking water source, the smoking prevalence, and the prevalences of additional demographic variables of the county’s population. The same outliers were excluded.

### 3.4. Restricted Poisson Log-Linear Model

The purpose of these restricted subset analyses is to examine the dose-response relationships for lung cancer incidences for populations with a reasonable degree of exposure and known to be in compliance with the ≤50 μg/L arsenic standard. Table 1 reveals a full range of dependency on groundwater wells as drinking water sources. The median dependency was 87% with a mean dependency of 74%. Of the 757 counties, 410 (54%) had a groundwater well dependency of 80% or greater for their drinking water supply.

**Figure 3:**
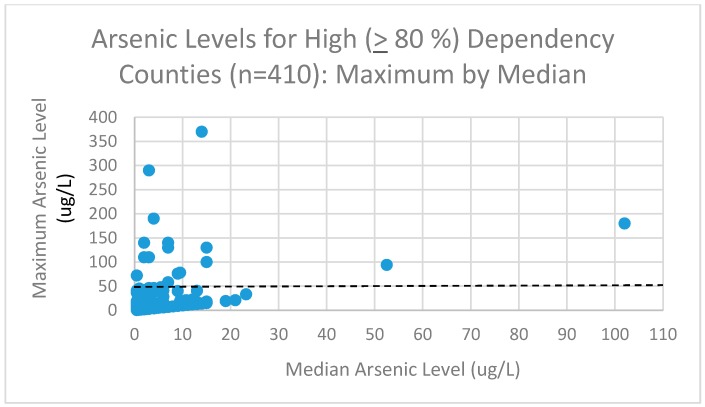
Maximum arsenic level by median arsenic level for high dependency (>80%) counties.

Figure 3 shows the high (≥80%) dependency counties (*n* = 411) with their maximum and median arsenic levels.

It is observed that some (*n* = 16) of the counties had individual wells that were out of compliance with the ≤ 50 μg/L standard. It is noteworthy that 10 of the counties with median arsenic levels ≤15 μg/L had wells with arsenic levels of 100–370 μg/L and another four had wells with arsenic levels of 50–99 μg/L. The lung cancer risks of these counties would not be related to drinking water that was in compliance with the 50 μg/L arsenic standard.

In order to examine the association between low levels of arsenic in the drinking water and lung cancer incidence for counties with a major dependency on drinking water, we have chosen to limit or restrict the analysis first to those 410 counties for whom at least 80% of its residents were dependent on the groundwater wells as the source for their drinking water, and then secondly to those 394 counties among them that were known to have all wells at ≤50 μg/L arsenic. That also eliminates the two counties with median arsenic levels ≤25 μg/L. These 394 counties with dependency ≥80% and maximum arsenic levels <50 μg/L serves as our restricted dataset for analysis. The population of these 394 counties comprised 11% of the 2010 US total population (34.8 M/308.7 M = 38%) and more than 56 million person-years of observation. These counties were in 36 states and distributed within the US.

After restricting our analyses to those 394 counties with high (≥80%) groundwater dependency and low (≤50 μg/L) maximum arsenic exposures, we developed Table 3.

Table 3 shows, in analyses restricted to the high dependency (≥80%) and low arsenic exposure (≤50 μg/L) counties (*n* = 394), the estimated coefficients from the unadjusted and adjusted models for total, male, and female populations. The coefficient for the median arsenic level was highly significantly negative (*p* < 0.001) in each of the three unadjusted restricted models and significantly negative in each of the three adjusted restricted models (Total, *p* < 0.001; Male, *p* = 0.001; Female, *p* = 0.029). Back-step regression showed no change in the coefficients for the median arsenic levels.

These analyses present a consistent finding of statistically significant negative coefficients for the lung cancer incidence risk versus median arsenic level in all counties with high groundwater dependency (≥80%) and low arsenic level (i.e., Maximum ≤ 50 μg/L).

### 3.5. Sensitivity Analysis

The three primary restrictions or assumptions in the analysis above were restricting the analysis to those counties all of whose groundwater drinking well arsenic levels were ≤50 μg/L and whose groundwater dependency was ≥80% when using the median arsenic value as the exposure metric. We now investigate how the primary association changes might have been influenced by such restrictions. Table 4 shows the result of restricting the maximum value to ≤100 μg/L instead of to ≤50 μg/L.

Table 5 shows the result of expanding the groundwater dependency window from ≥80% to ≤50%.

Table 6 shows the result of changing the exposure metric from the county-specific median arsenic level to the mean arsenic level and analyzing it as the natural logarithm of the mean (Ln Mean).

The number of counties included and excluded in the Table 4, Table 5 and Table 6 models change as the underlying filtering assumptions change. However, in each table the coefficients and other results are similar across the three study populations and all remain statistically significant and negative. This suggests that our findings of statistically significant negative associations are robust to the variety in the choice of the subset of available counties. These results are contrary to the prior expectation that any statistically significant slope (i.e., significantly different from zero) would have been positive rather than negative.

### 3.6. Stratified Risk Analysis

The EPA arsenic standards have been at ≤50 μg/L and at ≤10 μg/L. Stratified analyses at these levels may give an indication of the efficacy of these exposure limits with respect to the lung cancer risk at no-detectable levels of arsenic (i.e., <1 μg/L), at least for lung cancer. Table 7 shows the results of the stratification of the counties with dependency ≥80% and maximum ≤50 μg/L into those three exposure strata (median <1 μg/L; 1–10 μg/L, and >10–50 μg/L) and the analysis with the adjusted log-linear model.

For all three populations, the lung cancer risk is significantly lower for those counties with a median arsenic level of 10–50 μg/L, i.e., between the old and the new standard, than for counties with no arsenic detected in the drinking water. For both the total and male populations, but not for the female population, the lung cancer risk is significantly lower for those counties with a median arsenic level of 1–10 μg/L, i.e., in compliance with the new standard, than for counties with no arsenic detected in the drinking water. These analyses suggest caution that the assumption that lower exposure levels are better levels may not always be true.

## 4. Discussion

Although the MCL for arsenic concentration in drinking water has historically been ≤50 μg/L for over 70 years, it has not previously been assessed for its efficacy as a preventative against excess lung cancers. We have here examined lung cancer incidence in US counties whose drinking water supply from groundwater were all measured at less than or equal to 50 μg/L. We have used as our exposure metric the median arsenic level aggregated at the county level and derived our outcome metric from the 2009–2013 (5-year) lung cancer incident rate, also aggregated at the county level. Our log-linear regression analytic models yielded coefficients that were both negative and statistically significant with similar results in models of the total, male, and female populations. These results are contrary to the usually anticipated positive association but had been predicted for this exposure range in a recent meta-regression analysis [25]. 

### 4.1. Literature Review

Our results are consistent with the available literature for lung cancer incidence at low arsenic levels, i.e., in the ≤50 μg/L to the ≤100 μg/L range. The following are found in the literature, including in two recent meta-regression analyses [26,27].

In Table 8 above, the obtained relative risk in each study is reported. Of the 16 relative risk estimates in the table, 11 are less than 1.0, two are 1.0, and three (primarily, the male smokers from Bangladesh) are between 1.2 and 1.4. None of the relative risks for lung cancer were statistically significant (i.e., *p* < 0.05), neither those above or those below 1.00. 

### 4.2. Analogous Studies

Ours is not the first study to examine the dose-relationship between low level arsenic exposure and lung cancer risk using US county data, but ours is the first lung cancer study to restrict itself to the analysis of counties with individual well arsenic levels all ≤50 μg/L, the historical limit. The two prior studies, [7,32], also used the USGS National Water Information System (NWIS) data for their exposure assessments and the NCI lung cancer data for their outcome measures. Neither examined the distribution of arsenic levels within the counties, and neither selected for counties with maximum arsenic levels of ≤50 μg/L. 

Ferdosi used USGS-assigned median arsenic concentrations from potable groundwater well sources for each county and limited their analyses to counties that had no surface water sources for drinking water [8]. Their lung cancer data came from a 1983 NCI/US EPA study of 1950–1979 cancer mortality of US counties.

The mortality coefficients (slopes) in the Ferdosi study were based on standardized mortality ratios (SMRs) and for county median arsenic levels over the range of 3–59 μg/L. The overall slopes for the males and for the females were both negative, but not statistically distinguishable from zero. However, in stratified analyses, a statistically significant reduced relative risk of 0.97 for males and 0.98 for females were observed over the range of 10–59 μg/L and non-significant relative risks (1.01 for males and 1.01 for females) were observed over the range of 3.1–9.9 μg/L, compared to counties with a median of 3.0 μg/L arsenic [8]. The Ferdosi US county lung cancer mortality study [8] had been modeled after the earlier analogous Lamm US county bladder cancer mortality study [32]. 

The Mendez study was an update of the Lamm bladder cancer mortality study and the Ferdosi lung cancer mortality study but with reference to 2006–2010 bladder and lung cancer incidence cases rather than 1950–1979 mortalities. Mendez used mean arsenic levels rather than median arsenic levels as the exposure metric for the counties [7]. The earlier studies had restricted analysis to counties whose full public drinking water supply came from the groundwater wells [8,32]. The Mendez study restricted their analysis to counties whose populations had at least a 10% chance of having used the local drinking water sources, i.e., for whom up to 90% of the resident population may not have been exposed [7].

At the time of the earlier studies, arsenic exposure and cancer outcome data were available from all states and US EPA regions. At the time of the more recent studies, Mendez and ours, data from some states and one US EPA region were considered to be proprietary and not available to the public but were available to governmental agencies. Thus, those data are included in the analyses by Mendez and his colleagues from the US EPA but are not included in our analyses. 

Mendez reported for lung cancer that the incidence coefficient (slope) was statistically significantly positive for females for county mean arsenic levels with a range up to 158 μg/L and remained statistically significantly positive through a variety of sensitivity analyses. In contrast, their reported incidence coefficient for males was generally non-significantly negative and became significantly negative (*p* = 0.023) after potentially influential observations were omitted [7].

Our study was undertaken as a follow-up of the Ferdosi study with the intent of reporting on lung cancer incidence rather than on lung cancer mortality, of using recent outcome data that allowed for a decent latency period from the exposure data, and on focusing upon counties whose exposure levels were all within the historic arsenic drinking water limit of ≤50 μg/L. 

Following the publication of the Mendez study, we expanded our covariates to include their additional measures of drinking water well dependency and of obesity prevalence and attempted to replicate the Mendez findings. We were unable to replicate the positive slopes they found for the female lung cancers. We speculate that their finding may have been dependent upon the proprietary, non-public data or their non-restriction to low-level wells. 

We do know that a number of their county mean arsenic levels were grossly influenced by the inclusion of wells with arsenic levels in the several hundreds of μg/L. Extreme examples are those of Washington County, Idaho (FIPS 16087) and Worchester County, Massachusetts (FIPS 25208) Washington County, Idaho (FIPS 16087) had 16 wells with a mean arsenic level of 79 μg/L, a maximum of 950 μg/L, and a median of 10.5 μg/L. Removal of the singularly high level would bring the mean down to 21 μg/L, thus demonstrating lack of robustness in exposure assessment. Similarly, Worcester County, Massachusetts (FIPS 25208) had 57 different wells with a mean arsenic level of 38.7 μg/L (i.e., ≤50 μg/L), a maximum of 950 μg/L and a median of 2 μg/L. Eleven of its wells had arsenic levels >50 μg/L. These and similar counties with high arsenic level wells would not have been in our analysis.

**Figure A1:**
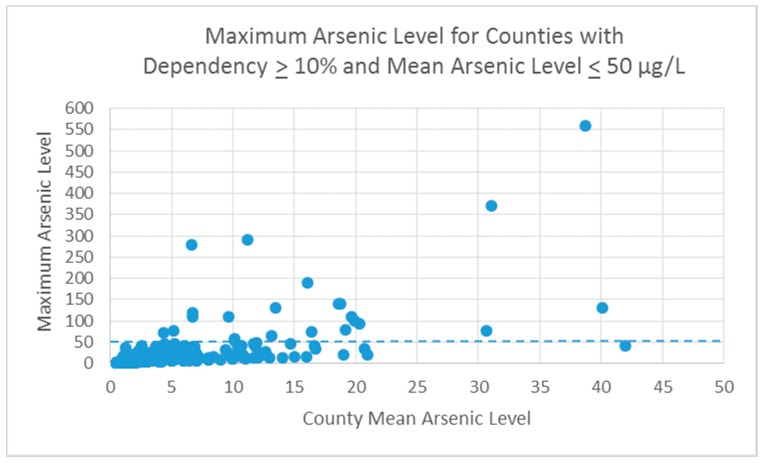
County drinking water maximum arsenic level by county drinking water mean arsenic level for counties with dependency ≥10% and mean drinking water arsenic level <50 μg/L.

For example, when we attempted to replicate their sensitivity analysis which had been restricted to counties with a mean arsenic level ≤50 μg/L, we found it still included counties with very high arsenic levels. In our dataset, we found multiple such counties (*n* = 23) that had individual well arsenic levels >50 μg/L in spite of a mean arsenic level ≤50 μg/L. (Appendix A Figure A1). These counties had an average maximum exposure of 147 μg/L and a range of 52–560 μg/L. It is clear that the cut-off of mean arsenic at ≤50 μg/L did not limit the analysis to counties that only had low arsenic levels, i.e., a maximum well arsenic level of ≤50 μg/L.

In our attempts to replicate the Mendez findings, we performed the multivariate analysis using the additional Mendez assumptions (Adjusted Poisson log-linear Model with Ln Mean Arsenic and ≥10% dependency). We found statistically significant negative coefficients for the total (Coef = −0.0085; *p* < 0.001) and the male populations (Coef = −0.0208; *p* < 0.001) and a non-significant negative coefficient for the female population (Coef = −0.002; *p* = 0.528). (Appendix A Table A1). Further, in order to analyze a “cleaner” dataset, we replicated our analysis by eliminating the data we had from the 96 counties in the nine states which had “proprietary” arsenic data. The results were no different (Total: Coef = −0.012, *p* < 0.001; Male: Coef = −0.0262, *p* < 0.001; and Female: Coef = −0.003, *p* = 0.500). (Appendix A Table A2). 

## 5. Limitations and Strengths

The two primary limitations of our study are those of any ecological study, i.e., that the data—exposure, outcome, and confounding variables—are all at the county level of data aggregation and not at the individual level, and that we are unable to account for long-term in- and out-migration for the counties. Further, we do not know the distribution of the county’s population as it relates to the distribution of the levels of arsenic in the drinking water in different wells supplying the county’s population.

An additional limitation is that we do not have specific information on the dietary, behavioral, and genetic characteristics of the study populations. However, we note that this is broadly a national study of the diverse US population with their range of variability in these factors. As we do not know the specific daily ingestion volumes and dietary arsenic intake, body sizes, etc., we have had to assume that they are characteristic of the general US population and accept general assumptions.

As the lung cancer incidence data did not provide cell-type specificity, we were unable to examine the finding of the Kuo study in Taiwan that only squamous cell carcinoma of the lung was associated with arsenic level in drinking water [33]. 

A strength of our ecological analysis is that we have been able to adjust our model at the county level not only for the major environmental causes of lung cancer—smoking—but also for the second major environmental cause of lung cancer—radon. We are also able to examine the issue of confounding between arsenic and cigarete smoking. Confounding is demonstrable with the statistically significant negative slope observed among the total restricted total, male, and female populations also being observed for those areas with smoking prevalence at or below the median but not for those with smoking prevalence above the median. At the higher smoking prevalences, the weaker arsenic risk is overwhelmed and not observed. 

Again, these are data that have been collected by governmental agencies for their purposes, and not specifically for the study at hand, and, as they are all publicly available data sets, our study can be replicated independently by other scientists.

### 5.1. Context of Exposure

Meacher et al. developed an estimation of the multimedia inorganic arsenic intake of the US population [34]. They concluded that while food is the greatest source of inorganic intake in the US population and that drinking water is the next highest contributor, regional differences in inorganic arsenic exposure were mostly due to consumption of drinking water containing differing inorganic arsenic content rather than food preferences. More recently, the Aylward study demonstrated that food was a major contributor to urinary arsenic levels in the US [35]. Inhalation of arsenic and ingestion of soil were negligible contributors. They reported that the mean of the distribution of tap water intakes for adults was 1.1 L and calculated the national mean amounts (μg) of inorganic arsenic intake, excluding water intake, to be 3.65 μg/day for males and 2.83 μg/day for females. Using these assumptions and the median arsenic levels, we have analyzed the lung cancer risk for each of all three populations and have found it to be statistically significantly negative for total and male populations and non-significantly negative for the female population both with respect to the estimated daily arsenic ingestion (μg/day) (Appendix A Table A3) and with respect to the estimated daily dosage (mg/kg/day), assuming additionally an average body weight of 70 kg (Appendix A Table A4).

Finally, there is the question as to how to express the summarization of the exposures of this group of counties. This group of counties which had arsenic levels of groundwater wells used for drinking water that were ≤50 μg/L and whose residents had a ≥80% groundwater dependency have in common that they have mean arsenic levels below 40 μg/L and median arsenic levels below 25 μg/L. So, the study population could alternatively be described as those with a dependency of ≥80% whose maximum drinking water arsenic levels did not exceed 50 μg/L, or whose mean drinking water arsenic levels did not exceed 40 μg/L, or whose median drinking water arsenic levels did not exceed 25 μg/L. Each is a true statement.

### 5.2. Lung Cancer Risk at Low Arsenic Exposure

The standard risk analyses expect to get a positive coefficient, whether over a broad range that includes both high exposure levels and low exposure levels or over a narrow range that only includes low exposure levels. The standard queries then are whether the association is statistically significantly positive and what is its magnitude. 

The Lamm meta-regression included all studies whose data extended across the broad range from low to high dosage [26]. They demonstrated for each of the six studies in their analysis a non-linear pattern with a downward curve at lower exposure levels and an upward curve at higher exposure levels. These data were fitted to a variety of non-linear models (polynomial through cubic, logistic, exponential, and power models) with the most consistent pattern being seen for the linear-quadratic model. The pattern was no different for the ecological mortality studies or the epidemiological incidence studies.

The analyses showed a statistically significant fit to a linear-quadratic model with a statistically significant negative linear function and a statistically significant positive quadratic function on a log-log plot. The X-intercept, the exposure level at which the risk returns to background, was at 136 μg/L (97–206 μg/L for a variety of models grouped by study design). This result was very similar to the inflection point at 127 μg/L that had been demonstrated by Lamm for lung cancer mortality in the SW Taiwan arsenic study [36]. These were the first analyses to predict that the slope for lung cancer might be significantly negative at low exposures. 

These findings of the linear-quadratic model suggested the stimulation by arsenic of both anti-carcinogenic processes that dominated at low-level exposures and pro-carcinogenic processes that dominated at high-level exposures.

The Lynch meta-regression included incidence studies that individually spread across the high to low range and included one mortality study by D’Ippolito that was limited to the low range [27,6]. They assumed a linear, no-threshold model for their analyses and found that their pooled cancer risk was driven by the high-arsenic data. Their US-only analysis, over the lower relative exposure range, yielded negative, non-significant associations. This conclusion, while not statistically significant, was consistent with that of Lamm meta-regression.

Our analysis demonstrates the observation that was predicted by the Lamm meta-regression, which is that the dose-response relationship for lung cancer incidence at low arsenic exposure is statistically significantly negative [26].

While this observation is noted here for lung cancer incidence, it should not be presumed to apply to other carcinogenic outcomes with arsenic ingestion, to non-carcinogenic outcomes with arsenic ingestion, or to outcomes associated with arsenic inhalation.

### 5.3. Toxicological Considerations

One view of the epidemiological data is that the arsenic-induced human cancers (skin, bladder, and lung) are seen at arsenic exposures in the few hundred of μg/L but not at the few tens of μg/L. Such an observation supports the concept of arsenic carcinogenicity as a non-linear threshold carcinogen, possibly a high-dose carcinogen. This concept has been discussed for decades, including by scientists from the US EPA in 1995 [37] and from the US FDA in 1998 [38]. There may be specific low-dose effects that are either anti-carcinogenic or pro-carcinogenic. 

Additional explanations derived from relevant epidemiological literature include the following: Chen associated arsenic-related cancers with blackfoot disease prevalence in southwest Taiwan and with the use of artesian wells [39]. Lamm proposed that the artesian well association might be explained as either arsenic acting as a high-dose carcinogen or as a co-carcinogen with some other aspect of artesian well water, possibly humic acid [40]. Tsuji had shown in a meta-analysis of populations with arsenic concentrations largely <100 μg/L (i.e., low-dose) that the non-significant positive slope for bladder cancer with arsenic ingestion was solely due to risk in smokers, as the slope for non-smokers was non-significantly negative [41].

The observations in this paper, as well as those in Ferdosi, support the “J” shaped curve found in the Lamm meta-regression and are consistent with the concept of hormesis and arsenic as discussed by Calabrese [8,26,42]. These epidemiological observations of a negative cancer slope factor at low arsenic doses and of a positive cancer slope factor at high arsenic doses is also consistent with the toxicological literature that has explored the modes of action of arsenic at various dose levels.

Snow demonstrated for human keratinocyte and fibroblast cells that low levels of arsenic (tissue levels of 0.1 to 1.0 μM) produced a protective effect against oxidative stress and DNA damage and, in contrast, that high levels of arsenic (tissue levels of >10 μM) induced down-regulation of DNA repair, oxidative DNA damage, and apoptosis [43]. Gentry also concluded that there were adaptive changes below 0.1 μM, adverse biological effects at 0.1 to 10 μM, and lethal effects above 10 μM [44]. Likewise, Cohen reported that it was only at high levels (>100 μM) that the severe cytotoxicity of the epithelial tissues (bladder, lung, and skin) was followed by regenerative proliferation leading to carcinogenesis of the urothelium, the bronchial epithelium, and the epidermis [45,46].

This pattern is not unique for arsenic. Dose-dependent transitions in mode of action from low to high exposure have been demonstrated for a number of carcinogens [47,48]. Most clearly, the modes of action for formaldehyde and nasal cancers in rats also show dose-dependent transitions with upregulation of protective enzymes at about 1 ppm, stimulation of an increase in DNA repair at 2 ppm, and initiation of apoptosis and pro-carcinogenic effects at 5–6 ppm [49].

The weight of the evidence, both in this study and in the epidemiological literature, seem to indicate a downward slope, a reduced risk, with respect to low-level arsenic ingestion and human lung cancer. This observation must be similarly examined for other human cancers and for non-cancer effects of arsenic exposure.

Arsenic is not the only environmental exposure considered here as a causal agent for lung cancer. These analyses consider simultaneously the effects of arsenic, smoking, and radon as explanatory exposure covariates. Poisson log-linear regression with and without the specified exposure covariates revealed that smoking made the greatest contribution, accounting for about 13% of the explanatory variation of the model. Arsenic exposure only accounted for about 1% of the explanatory variation, and radon exposure accounted for <0.1%. 

## 6. Conclusions

The objective of this ecological study was to assess the association between low arsenic levels in drinking water and the incidence of lung cancer in the US population using recent data aggregated at the county level. We defined low arsenic levels as having a maximum exposure level of ≤50 μg/L and populations with 80% or greater dependency on such waters. We found a statistically significant negative association (slope) for low levels of arsenic and lung cancer incidence of US counties for total, male, and female populations, and in both unadjusted and adjusted Poisson log-linear models. Each of these counties had a maximum arsenic level of 50 μg/L.

The analyses of the most relevant data (≥80% dependency and ≤50 μg/L) have yielded significant negative coefficients of −0.004 to −0.006 for this range of median arsenic exposures. These coefficients would convert to relative risks of 0.994 to 0.996, which seem small and possibly not of public health significance. However, that means that these data demonstrate that this exposure level here is not associated with an increased risk of lung cancer but rather is significantly associated with a decreased risk of lung cancer, approximately with a ½% reduction in risk per μg/L arsenic. Due to the small effect size, it has been necessary to have a very large study in order to have the power to detect a significantly negative comparative small effect. This analysis has included 11% of the US population that was observed over a five-year time period for a total of more than 56 million person-years of observation. This observation is limited to lung cancer incidence and may or may not be observed for other cancers or for non-cancer effects. 

## Figures and Tables

**Table 1:** Data characteristics of analytic variables for individual counties.

Variable	Mean	Median	Minimum	Maximum
Outcome				
Lung Cancer Rate				
(per 100,000)	66.3	66.3	13.5	124.8
Count (5-year estimate)	463	136	8	11,459
Exposure				
Dependency	74%	87%	0%	100%
Well Count	8.7	2	1	274
As Median (μg/L)	2.1	0.8	0.5	102
As Minimum (μg/L)	1.2	0.5	0.5	42
As Maximum (μg/L)	11.1	1	0.5	950
Variables				
Current Smoker (%)	24.3	24.7	6.2	40.7
Ex-Smoker (%)	23.1	23.4	10.6	37.4
Obesity (%)	30.3	30.9	13.9	44.6
Education (<HS)	0.15	0.14	0.03	0.34
Residency (Same Cnty)	0.94	0.94	0.81	0.99
Poverty	0.16	0.16	0.04	0.53
Income ($ K)	47.4	44.8	23.9	106.1
Rural	0.49	0.48	0.00	1.00
Population				
Male	0.50	0.50	0.45	0.64
Hispanic	0.09	0.04	0.00	0.82
White	0.87	0.92	0.03	0.99
Black	0.07	0.02	0.00	0.69
Asian	0.02	0.01	0.00	0.22
Other	0.05	0.02	0.01	0.97
Population	154,960	38,966	2389	4,092,459

**Table 2:** Unadjusted and adjusted Poisson log-linear models on lung cancer incidence.

Variable	Total	Male	Female
Unadjusted Median Model			
N	757	704	678
As(Median)	−0.007 ***	−0.009 ***	−0.006 ***
Intercept	−5.801 ***	−5.637 ***	−5.937 ***
	Total	Male	Female
Adjusted Median Model			
N	748	695	669
As (Median)	−0.001 *	−0.003 ***	−<0.001
GW Dependency	−0.025 ***	−0.030 ***	0.012
Current Smoker Prevalence	0.016 ***	0.017 ***	0.013 ***
Ex-smoker Prevalence	0.010 ***	0.008 ***	0.011 ***
Radon (>4 pCi/L)	−0.004	−0.003	−0.006
Obesity	0.004 ***	0.004 ***	−0.002 **
Education (≥high school)	−0.022 ***	−0.017 ***	−0.021 ***
Residency (same county, prior year)	−0.084	0.477 ***	0.165
Poverty (<poverty live)	−0.340 **	−0.689 ***	0.021
Median Household Income ($ K)	−0.001 *	−0.003 ***	0.0006
Rural	−0.288 ***	−0.214 **	−0.297 ***
Male (%)	−1.421 ***		
Hispanic	−0.896 ***	−0.831 ***	−1.029 ***
Black	−0.021	0.137 ***	−0.068 *
Asian	0.220 ***	0.311 ***	0.129
Other	−0.705 ***	−0.919 ***	−0.568 ***
Intercept	−3.488 ***	−4.823 ***	−4.408 ***

* *p* < 0.05; ** *p *< 0.01; *** *p *< 0.001.

**Table 3:** Unadjusted and adjusted Poisson log-linear models for lung cancer incidence restricted to US counties with high dependency (≥80%) and low maximum arsenic exposure (≤50 ug/L).

Variable	Total	Male	Female
Unadjusted Median Model			
N	394	351	334
As(Median)	−0.008 ***	−0.010 ***	−0.007 ***
Intercept	−5.803 ***	−5.625 ***	−5.951 ***
	Total	Male	Female
Adjusted Median Model			
N	393	350	333
As(Median)	−0.005 ***	−0.006 **	−0.004 *
GW Dependency	0.492 ***	0.659 ***	0.389 ***
Current Smoker Prevalence	0.021 ***	0.023 ***	0.020 ***
Ex-smoker Prevalence	0.015 ***	0.011 ***	0.016 ***
Radon (>4 pCi/L)	−0.003	−0.015	−0.008
Obesity	0.002	−0.001	−0.006 ***
Education (≥high school)	−0.022 ***	−0.016 ***	−0.022 ***
Residency (same county, prior year)	−0.069	0.052	0.337
Poverty (<poverty live)	−0.495 **	−0.749 ***	−0.123
Median Household Income ($ K)	<0.001	- <0.001	0.001
Rural	−0.286 ***	−0.198 ***	−0.280 ***
Male (%)	−1.224 ***		
Hispanic	−0.749 ***	−0.657 ***	−0.893 ***
Black	0.017	0.230 ***	−0.061
Asian	1.207 ***	0.822 ***	1.682 ***
Other	−0.962 ***	−1.207 ***	−1.016 ***
Intercept	−4.352 ***	−5.473 ***	−5.067 ***

* *p* < 0.05; ** *p* < 0.01; *** *p* < 0.001.

**Table 4:** Adjusted Poisson log-linear model with ≥80% dependency and maximum arsenic <100 μg/L.

Restrictions (GW Dependency ≥ 80%; Max ≤ 100 μg/L)					
Population	*n*	Coef	SE	z	*p*
Total	399	−0.004	0.001	−3.10	0.002
Male	359	−0.005	0.002	−3.54	<0.001
Female	338	−0.001	0.002	−0.63	0.531

**Table 5:** Adjusted Poisson log-linear model with ≥50% dependency and maximum arsenic ≤50 μg/L.

Restrictions (GW Dependency ≥ 50%; Max ≤ 50 μg/L)					
Population	*n*	Coef	SE	z	*p*
Total	513	−0.006	0.001	−5.05	<0.001
Male	515	−0.007	0.002	−4.51	<0.001
Female	496	−0.008	0.002	−4.563	<0.001

**Table 6:** Adjusted Poisson log-linear model with mean arsenic level, ≥80% dependency and maximum arsenic ≤50 μg/L.

Restrictions (Ln Mean Arsenic; GW Dependency ≥ 80%; Max ≤ 50 μg/L)					
Population	*n*	Coef	SE	z	*p*
Total	393	−0.026	0.005	−5.521	<0.001
Male	350	−0.036	0.006	−5.81	<0.001
Female	333	−0.019	0.007	−2.72	0.007

**Table 7:** Adjusted Poisson Log-Linear models of median arsenic level, stratified at 10 μg/L and at 50 μg/L and compared to <1 μg/L, for counties with ≥80% dependency and maximum arsenic ≤50 μg/L.

Population	Concentration	Ceof. *	SE	z	*p*-Value
Total	>10–50 μg/L	−0.088	0.020	−4.19	0.000
Male	>10–50 μg/L	−0.093	0.027	−3.46	0.001
Female	>10–50 μg/L	−0.066	0.032	−2.04	0.041
Total	1–10 μg/L	−0.045	0.008	−5.71	0.000
Male	1–10 μg/L	−0.093	0.010	−9.35	0.000
Female	1–10 μg/L	−0.017	0.012	−1.45	0.146

* compared to <1 μg/L.

**Table 8:** Low-arsenic and lung cancer incidence literature.

Reference (Year)	Location	μg/L	RR *
Dauphine et al. (2013) [6]	California/Nevada	42.5	0.75
Steinmaus et al. (2014) [28]	Chile	35	1.24
		80 ^a^	0.89
Steinmaus et al. (2013) [5]	Chile	52.5 ^b^	0.98
Ferreccio et al. (2013) [3]	Chile	60	0.77
Smith et al. (2009) [4]	Chile	12.8	0.7
		35 ^c^	0.7
Bogen et al. (2014) [29]	NE Taiwan	3.26	0.57
		25.9	0.73
		74.3	0.68
Chen et al. (2010b) ^d^ [30]	NE Taiwan	30	1.1
		75	0.99
Mostafa et al. (2008) [31]	Bangladesh males		
	Non-Smokers	30	0.9
		75	1.1
	Smokers	30	1.25
		75	1.37

* None with *p* < 0.05; ^a^ Steinmaus et al. (2014) [28]—Web Table 4, highest five years with mid-range level; ^b^ Steinmaus et al. (2013) [5] and Ferreccio et al. (2013) [3] are the same dataset with Steinmaus using the mid-point of lifetime average exposure strata of 26–79 μg/L and Ferreccio using the highest one-year exposure level; ^c^ From Smith et al. (2009) [4], Lamm et al. (2015) [25] cites the population-weighted exposure level of 12.8 μg/L and Lynch et al. (2017) [26] cites the mid-range level (35 μg/L) for the 10–59 μg/L strata; ^d^ Chen (2010b) [30] is the published version; Bogen (2014) [29] is a presented version with a revised reference population.

## Appendix A

**Table A1:** Adjusted Poisson log-linear model with ln mean arsenic, >10% dependency, and ND = 1/(sqrt 2) × LOD [Mendez model].

Restrictions (Ln Mean Arsenic; GW Dependency ≥10%; ND 1/(Sqrt 2))					
Population	*n*	Coef	SE	z	*p*
Total	735	−0.009	0.002	−4.40	<0.001
Male	682	−0.021	0.003	−7.33	<0.001
Female	657	−0.002	0.003	−0.63	0.528

**Table A2:** Adjusted Poisson log-linear model with ln mean arsenic, >10% dependency, and ND = 1/(sqrt 2) × LOD, excluding states that had supplementary data in the Mendez dataset. [Mendez model, excluding states with “proprietary” data].

Restrictions (Ln Mean As; NWIS; GW Dependency ≥10%; ND 1/(Sqrt 2))					
Population	*n*	Coef	SE	z	*p*
Total	639	−0.012	0.003	−5.04	<0.001
Male	595	−0.026	0.003	−8.09	<0.001
Female	571	−0.003	0.004	−0.67	0.500

**Table A3:** Adjusted Poisson log-linear model with daily arsenic dosage (μg/day), 1.1 L/day; ≥80% dependency, As(Max) ≤ 50, and ND = 1/2 LOD.

Restrictions (Daily Dosage; 1.1 L/day; GW Dependency ≥ 80%; As(Max) ≤ 50)					
Population	*n*	Coef	SE	z	*p*
Total	393	−0.004	0.001	−3.67	0.000
Male	350	−0.005	0.002	−3.06	0.002
Female	333	−0.003	0.002	−1.80	0.071

**Table A4:** Adjusted Poisson log-linear model with arsenic dose rate (mg/Kg/day), 1.1 L/day, ≥80% dependency, As(Max) ≤ 50, and ND = 1/2 LOD.

Restrictions (Dosage [mkd]; 70 kg; GW Dependency > 80%; As(Max) ≤ 50)					
Population	*n*	Coef	SE	z	*p*
Total	393	−304.4	82.9	−3.67	0.000
Male	350	−329.1	107.5	−3.06	0.002
Female	333	−226.3	125.6	−1.80	0.071
